# Dysbiosis of urine microbiota in obstructive urinary retention patients revealed by next-generation sequencing

**DOI:** 10.1186/s12941-020-00408-5

**Published:** 2021-01-06

**Authors:** Shan Jiang, Saisai Lu, Xiaomin Chen, Fengxia Li, Chengwei Zhu, Yuancai Zheng, Xiaobing Wang, Shihao Xu

**Affiliations:** 1 Institute of Genomic Medicine, Wenzhou Medical University, Wenzhou, China; 2 Department of Rheumatology, The First Affiliated Hospital of Wenzhou Medical University, Wenzhou, China; 3 Department of Urology, The First Affiliated Hospital of Wenzhou Medical University, Wenzhou, China; 4 Department of Ultrasonography, The First Affiliated Hospital of Wenzhou Medical University, Wenzhou, China

**Keywords:** Urine microbiome, Obstructive urinary retention, 16S rRNA gene sequencing

## Abstract

**Background:**

Urinary retention (UR) is a common urinary system disease can be caused by urinary tract obstruction with numerous reasons, however, the role of urine microbes in these disorders is still poorly understood. The aim of this study was to identify the urine microbial features of two common types of obstructive UR, caused by urinary stones or urinary tract tumors, with comparison to healthy controls.

**Methods:**

Urine samples were collected from a cohort of 32 individuals with stone UR, 25 subjects with tumor UR and 25 healthy controls. The urine microbiome of all samples was analyzed using high-throughput 16S rRNA (16S ribosomal RNA) gene sequencing.

**Results:**

We observed dramatically increased urine microbial richness and diversity in both obstructive UR groups compared to healthy controls. Despite different origins of UR, bacteria such as *Pseudomonas, Acinetobacter* and *Sphingomonas* were enriched, while *Lactobacillus*, *Streptococcus*, *Gardnerella*, *Prevotella* and *Atopobium* were decreased in both UR groups in comparison with healthy controls, exhibited an approximate urine microbial community and functional characteristics of two types of obstructive UR. Furthermore, disease classifiers were constructed using specific enriched genera in UR, which can distinguish stone UR or tumor UR patients from healthy controls with an accuracy of 92.29% and 97.96%, respectively.

**Conclusion:**

We presented comprehensive microbial landscapes of two common types of obstructive urinary retention and demonstrated that urine microbial features of these patients are significantly different from that of healthy people. The urine microbial signatures would shed light on the pathogenesis of these types of urinary retention and might be used as potential classification tools in the future.

## Background

Urinary retention (UR) is a series of complex diseases defined as the inability to completely empty the bladder due to various causes [1–3]. UR affects both men and women; however, it presents with a male to female ratio of 10:1 [4] due to the high presence of prostate gland problems in males [5]. UR can be classified into nonobstructive and obstructive types according to the cause of its formation. Nonobstructive causes include medications, bladder muscle weakness and nerve problems that interfere with the brain’s ability to receive signals that the bladder is full and the body’s ability to function properly [6–8]. Conditions such as benign prostatic hyperplasia (BPH), urinary tract stones and certain tumors can lead to urethral stricture or deformation cause obstruction [9]. UR can also be chronic or acute. Chronic UR can be a long-lasting and less painful medical condition, still resulting in urinary incontinence (UI), a urinary tract infection (UTI), and so on. In contrast, acute UR occurs suddenly and causes great discomfort or pain to the patient. It is a potentially life-threatening medical condition that requires immediate emergency treatment, such as bladder drainage, urethral dilation or surgery.

Increasing studies have revealed that bacteria not only exist in the urine tracts of healthy individuals but play a crucial role in the maintenance of microecological balance [10–13]. In addition, with the advent of culture-independent methods for detect microbe, amount of studies proved that the abnormal urine microbial community is also closely related with diverse urologic disorders. UTI accounts for a high proportion of these population, with the most common microbe being *Escherichia coli* followed by some gram-positive cocci and other Enterobacteriaceae [14]. In 2014, Meghan et al. used the expanded quantitative urine culture (EQUC) techniques observed that nine bacteria (*Actinobaculum, Actinomyces, Aerococcus, Arthrobacter, Corynebacterium, Gardnerella, Oligella, Staphylococcus, and Streptococcus*) were obviously exited in the urine of Urgency urinary incontinence (UUI) patients [15]. This team also revealed that the UUI urine microbiome consisted of increased *Gardnerella* and decreased *Lactobacillus* compared to the non-UUI group based on 16S rRNA gene sequencing [15]. Recent work by Siddiqui et al. found that more than 90% of sequencing reads in the urine of interstitial cystitis (IC) patients were belong to *Lactobacillus*, while 60% in healthy female urine [16]. Sequencing of bacteria-specific 16S rDNA in the mid-stream urine of 25 chronic prostatitis/chronic pelvic pain syndrome (CP/CPPS) patients and 25 asymptomatic or only had urinary symptoms men controls, demonstrated significantly higher phylogenetic diversity in the urine microbiota of CP/CPPS patients and higher *Clostridia* count than the control group [17]. Dornbier et al. sequenced 16S rRNA of bladder urine and urinary stones in 52 patients, suggested that the presence of bacterial communities in non-struvite stone was relatively higher than the surrounding urine [18]. Another study recruited patients with different stone type found that there was a common imbalance between the microbiome of urinary stone disease (USD) and different pathologies [19]. In addition, a cross-sectional study investigated the urine microbiome of asymptomatic bacteriuria who had risks from a neurogenic bladder due to spinal cord injury and healthy controls, showed altered abundance of *Lactobacillales*, *Enterobacteriales* and other microorganisms and confirmed functional interactions between pathogens and human proteins in subjects who initiated host defense [20].

Based on the above information, microorganisms have been widely studied in urinary system disease, but the potential relationship between the urine microbiome and obstructive UR has not yet been fully elucidated. Therefore, we performed next-generation sequencing of 16S rRNA to detect the urine microorganisms and their associated functional profile of two common obstructive UR, which were caused by stones or tumors, with comparison to that of healthy individuals. Disease classifiers were further constructed for patients with stone UR and tumor UR based on their specific microbial features.

## Methods

### Study population

All patients with obstructive UR and healthy controls were recruited from July 2018 to April 2019 at The First Affiliated Hospital of Wenzhou Medical University (Wenzhou, China) used for this study. Signed informed consent was obtained from each participant, for which all procedures and protocols were approved by the Medical Ethical Committee of the Wenzhou Medical University Ethics Committee. Two types of obstructive UR patients in our study were enrolled: one group presented with urinary stones, and the other group presented with obstructive UR caused by certain tumors in the urinary system that resulted in an inability to urinate automatically. The number of cases in the two groups was 34 and 25, respectively. Patients who had taken antibiotic or probiotic treatment in the past 8 weeks were also excluded. Twenty-five healthy individuals volunteered as our control group, none of whom had a history of UTI or urinary system disease. 30 ml fresh urine samples from all participants (n = 84) were collected by sterile catheter drainage under the strict aseptic procedures and treated in the same method after sampling, frozen in a sterile container at − 80 °C within 12 h after sampling.

### Biochemical indicator detection and routine urine analysis

Blood samples were collected from all subjects to measure biochemical metabolic parameters. Approximately 3 ml of whole blood was obtained from each volunteer’s blood. Plasma samples for all analyses were obtained by centrifugation for 15 min at room temperature at 3000 rpm and then frozen at − 80 °C for further analysis. Baseline biochemical indicators, including serum direct bilirubin (DBIL), indirect bilirubin (IBIL), eGFR, alkaline phosphatase (ALP) and UA, were assessed quantitatively with an automatic biochemical analyzer. Red blood cell count (RBC), white blood cell count (WBC), urobilinogen and proteinuria in urine samples were detected by routine urine analysis. A positive test is represented with at least one “+” sign in the column of urobilinogen or proteinuria value in the inspection report. All these clinical values were acquired based on standard procedures.

### DNA extraction and 16S rRNA gene amplicon sequencing

Bacterial DNA extraction from these urine samples was performed with a TIANamp Stool DNA Kit according to the manufacturer’s instructions. To distinguish the contaminants and existing microbial community, an unused clean sterile catheter was considered a negative control of sampling environment and a DNA extraction-negative control with no urine added.

The V3–V4 region of 16S ribosomal RNA from the extracted DNA samples was amplified with a universal forward primer (5′-CCTACGGGNGGCWGCAG-3′) and reverse primer (5′-GACTACHVGGGTATCTAATCC-3′) with a single multiplex identifier and adaptors. To exclude contamination during amplification, a PCR-negative control with no template DNA were processed. PCR amplification was then parallel performed in 25 μl of 2 × Phanta Max Master Mix, 2 μl of forward primer (10 μM), 2 μl of reverse primer (10 μM), 50 μl of ddH2O and template DNA. The PCR was conducted under the following conditions: 95 °C for 3 min; 25 cycles of 95 °C for 30 s, 55 °C for 30 s, and 72 °C for 30 s; and a final extension at 72 °C for 5 min. Sample library and Phix sequencing control library were added, and the sequencing reaction was performed on an Illumina MiSeq sequencer and yielded 300 bp paired-end reads at high depth.

### Bioinformatic analysis

To improve the quality of analytical data, this process utilizes the quality control section from our house pipeline [21, 22], including to remove those sequences that did not contain primers, ambiguous reads, and reads with an average quality value < Q20. Only sequences with a length longer than 300 bp and two reads with an overlap of more than 10 bp were merged using PANDAseq (v.2.9) [23]. The Quantitative Insights Into Microbial Ecology (QIIME) platform (v.1.9.1) [24] was used to pick the closest reference operational taxonomic unit (OTU) at a 97% similarity cut-off, and taxonomy assignment was then mapped using the Greengenes database (v.13.8) [25]. Samples were excluded if their total reads that can be annotated to OTUs were less than 8000, and OTUs with a number of sequences less than 0.01% of the total number of sequences were also discarded. In addition, rarefaction was performed with USEARCH [26], which randomly sampled 10–100% of the sequences from the original sequencing data to observe the number of OTUs annotated in each sequence set. Based on the OTU profile, the α-diversity was measured using the taxa richness and diversity, and the β-diversity was estimated by computing unweighted and weighted UniFrac [27] distances and Bray–Curtis dissimilarity and further visualized with principal coordinate analysis (PCoA). Linear discriminant analysis (LDA) for effect size (LEfSe) program were performed based on the web services tools Galaxy (http://huttenhower.sph.harvard.edu/galaxy/root/index) [28]. The features that were significantly different among the three groups were identified by LEfSe with P < 0.05 (Kruskal–Wallis test) and LDA values > 4 [29]. Phylogenetic Investigation of Communities by Reconstruction of Unobserved States (PICRUSt) (http://picrust.github.io/picrust) [30] predicted the gene family abundance from the phylogenetic information with an estimated accuracy at 0.8, which could be used to impute the urine microbiome metagenome from the 16S rRNA sequences. We selected level 3 Kyoto Encyclopedia of Genes and Genomes (KEGG) database pathways and level 2 Cluster of Orthologous Groups (COG) of abundance from the predicted functional profiles and filtered the “Function Unknown” and “Other” pathways. Only the functions and pathways with an average relative abundance > 0.01 that existed in at least ten samples were considered in the analysis.

### Statistical analysis

All statistical analyses were performed by R packages (version 3.4.3). The permutational multivariate analysis of variance (PERMANOVA) test was performed using the [Adonis] function of the “vegan” R package, with the maximum number of permutations = 999. For comparison of all continuous variables, community diversity and richness, significantly different OTU abundance and significantly different taxon abundance at phylum and genus levels were selected using the Wilcoxon rank sum test method as previously described [31, 32]. The resulting p-values were adjusted by the Benjamini and Hochberg false discovery rate (FDR). The correlation between different genera was calculated with a Spearman correlation based on the relative abundance, which was also used to study the relationship between different bacteria and clinical indicators. For the significantly different genus abundance profile, five-fold cross-validation was performed five times on a random forest model (‘randomForest’ 4.6-12 package). We then obtained the average cross-validation error curve from the five processed trials. The point with the minimum cross-validation error plus the standard deviation (SD) at the corresponding point was viewed as the cut-off point. We listed all sets of biomarkers with an error less than the cut-off value and chose the set with the smallest number of genera as the optimal set. To evaluate the discriminatory ability of the random forest model, we obtained the average area under the curve (AUC) and constructed the receiver operating characteristic curve (ROC) 100 times using the ‘ROCR’ R package. The mean decrease accuracy (MDA) was assigned to each feature based on the fact that removing the feature from the prediction model would increase the error rate. The probability of disease (POD) value refers to the ratio between the number of randomly generated decision tress that predicting an individual as a patient with stone UR or tumor UR and that of healthy controls. Similarly, the probability of tumor UR index is a predictive comparison between the two types of obstructive UR. The detailed script of the microbial optimal set identification and 100 times ROC analysis and POD construction can be found in the supplementary method and refer to the research published by Ren et al. in 2019 [33]. To explore which bacteria are closely related to the main enrichment functions and pathways from each group, the Spearman correlation method was also adopted.

## Results

### The OTU profile was obtained after 16S rDNA data analysis

To study the urine microorganisms in obstructive UR, we performed 16S sequencing on urine samples from 84 Chinese participants, including 34 patients with UR caused by lithiasis (stone UR), 25 patients with UR due to the urinary tract tumors (tumor UR), and 25 healthy controls. These negative controls were not sequenced, as the agarose gel electrophoresis showed that no electrophoretic band of microbial DNA, which indicates that there was no artifact and contaminant in the environment. A total of 727 MB of 300 bp paired-end reads were generated after sequenced on the MiSeq platform, and the average number of reads per sample was 36,072 ± 3376 reads (Additional file 1: Table S1). Two samples from the stone UR group, S18 and S32, were removed for their low assigned microbial reads, which were less than 8000. Finally, we obtained 310 OTUs from 25 controls, 32 stone UR and 25 tumor UR individuals (Additional file 2: Table S2). The clinical and demographic characteristics of all remaining UR patients and controls are shown in Table 1. In terms of age, there was no significant difference between the stone UR group and control group, while the tumor UR group subjects were older than the other two groups. There was no statistically significant difference in gender among these three groups.

**Table 1:** Clinical characteristics of the enrolled participants

Clinical indexes	Control (n = 25)	Stone UR (n = 32)	Tumor UR (n = 25)	*P*-value Control vs Stone UR	*P*-value Control vs Tumor UR	*P*-value Stone UR vs Tumor UR
Age (year)	42.92 ± 20.15	52.94 ± 14.40	61.60 ± 13.24	0.054	0.0013	0.034
Gender						
Female	17 (68%)	21 (65.62%)	16 (64%)	1	1	1
Male	8 (32%)	11 (34.38%)	9 (36%)			
DBIL (μmol/L)	2.50 ± 0.70	4.14 ± 2.74	4.56 ± 5.61	0.030	0.25	0.49
IBIL (μmol/L)	4.36 ± 1.85	6.53 ± 2.67	5.46 ± 2.76	0.043	0.58	0.12
eGFR	97.56 ± 23.70	70.63 ± 32.19	40.30 ± 33.32	0.013	0.00010	0.0032
ALP (μ/L)	91.08 ± 30.99	91.70 ± 33.98	144.92 ± 234.24	0.96	0.79	0.81
UA(μmol/L)	317.20 ± 54.00	367.96 ± 103.13	454.01 ± 160.95	0.24	0.039	0.063
RBC (/μL)	9.45 ± 10.60	691.23 ± 1348.03	3489.10 ± 6187.54	0.0015	0.068	0.56
WBC (/μL)	55.45 ± 123.05	2110.97 ± 5085.15	1472.82 ± 3973.84	0.0096	0.0026	0.59
pH						
> 6.5	2 (8%)	12 (37.5%)	7 (28%)	0.27	0.68	0.56
≤ 6.5	9 (36%)	17 (53.1%)	16 (64%)			
Urobilinogen						
Positive	1 (4%)	3 (9.4%)	2 (8%)	1	1	1
Negative	10 (40%)	27 (84.4%)	20 (80%)			
Proteinuria						
Positive	0 (0%)	20 (62.5%)	16 (64%)	0.00016	0.000086	0.76
Negative	11 (44%)	10 (31.2%)	6 (24%)			

DBIL: Direct bilirubin; IBIL: Indirect bilirubin; eGFR: estimated glomerular filtration rate; ALP: Alkaline phosphatase; UA: uric acid; RBC: red blood cell; WBC: white blood cell

### Increased urine microbial diversity in stone UR and tumor UR individuals

**Fig. 1:**
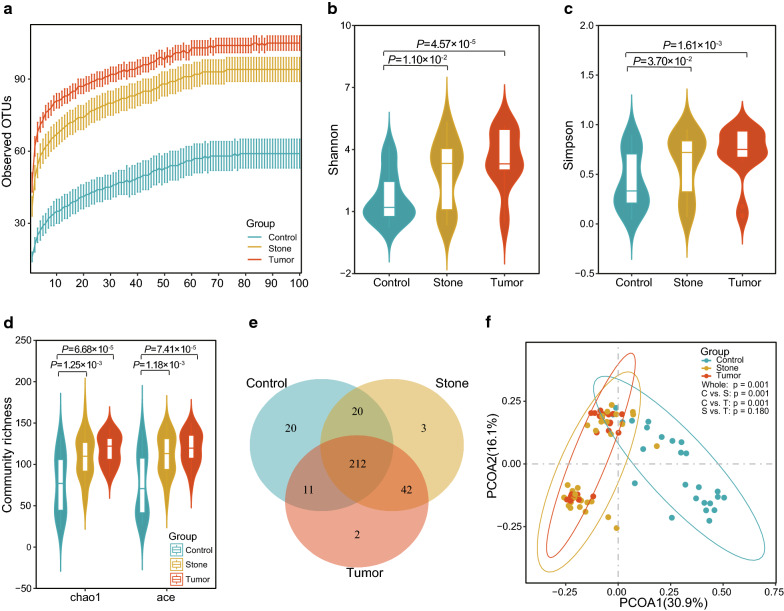
Increased urine microbial diversity of stone UR and tumor UR compared with that of controls. **a** Rarefaction curves for observed OTU number among three groups after random sampling of 10–100% sequences from the original sequencing data. **b** Shannon index and **c** Simpson index of the urine microbiome from stone UR and tumor UR and controls. **d** The Chao1 and Ace indices and community richness were estimated among three groups. **e** A Venn diagram exhibiting the shared and unique OTUs among three groups. **f** PCoA of unweighted UniFrac distance is depicted for UR and healthy controls

To depict the bacterial richness of each group, we randomly sampled the same amount of reads from each sample and performed rarefaction analysis to estimate the observed OTUs that could be identified from these sequences. As shown in Fig. 1a, all three curves had reached plateaus, which indicated that the amount of sequenced data were sufficient to detect the microbial feature. The acquisition rate of OTUs in control samples was strikingly lower than that in stone UR and tumor UR groups. Measured by the Shannon index, the urine microbial diversity of the stone UR and tumor UR was significantly greater than that of healthy controls, however, there was no significant difference between the two UR groups (Fig. 1b, Shannon: controls vs stone UR, P= 1.10 × 10^−2^; controls vs tumor UR, P= 4.57 × 10^−5^; stone UR vs tumor UR, P= 0.29). Likewise, the Simpson index of stone UR and tumor UR was significantly higher than that of controls (Fig. 1c, Simpson: controls vs stone UR, P = 3.70 × 10^−2^; controls vs tumor UR, P = 1.61 × 10^−3^; stone UR vs tumor UR, P = 0.28). The Chao1 index and Ace index, which measure the richness of the community, showed that the richness of microbiome in two UR groups was significantly higher than that in controls (Fig. 1d, Chao1: controls vs tumor UR, P= 1.25 × 10^−3^; controls vs tumor UR, P= 6.68 × 10^−5^; stone UR vs tumor UR, P= 0.15; Ace: controls vs tumor UR, P= 1.18 × 10^−3^; controls vs tumor UR, P= 7.41 × 10^−5^; stone UR vs tumor UR, P= 0.19). In addition, a Venn diagram (Fig. 1e) was used to characterize the overlapped OTUs among the three groups. A total of 212 OTUs were shared among the 3 groups, and there were 20, 3, and 2 OTUs were unique to the controls, stone UR and tumor UR groups, respectively. It is worth noting that there were as many as 42 OTUs shared between the stone UR and tumor UR groups.

To further investigate whether there were differences among the three groups in the urine microbiota spectrum, PCoA was performed based on the unweighted UniFrac distances of the 16S rRNA sequence at the OTU level. There were differences in β-diversity among the three groups, as shown in Fig. 1f (PERMANOVA, pseudo-F statistic: 11.88, P= 1.00 × 10^−3^). In addition, differences between each of the two groups were further evaluated based on unweighted and weighted UniFrac distances and Bray–Curtis dissimilarity (Additional file 3: Table S3). These results showed that both UR groups were significantly different from the control group, while the microbial compositions of two types of obstructive UR could not be separated.

### Altered urine microbial communities in stone UR and tumor UR

**Fig. 2:**
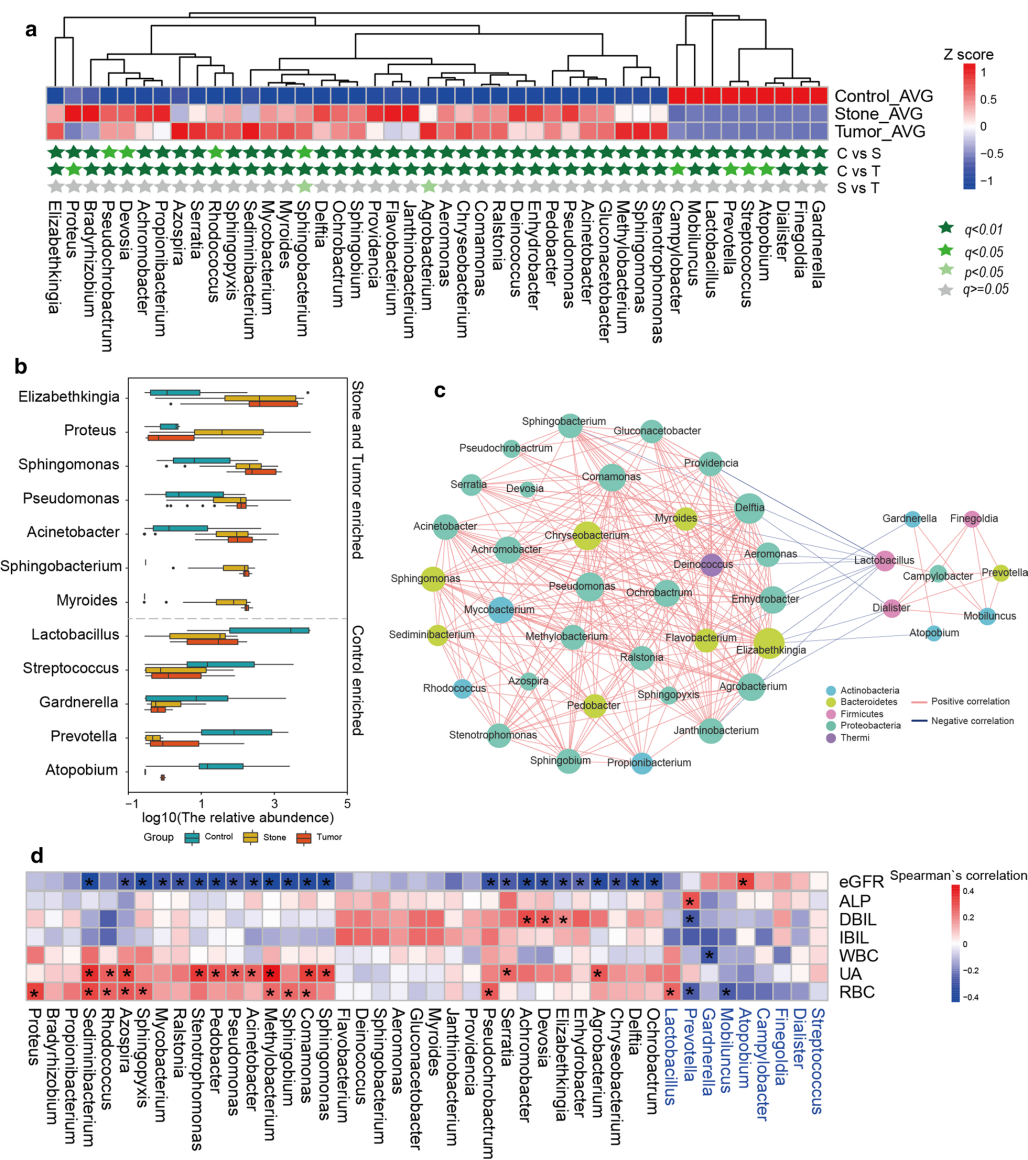
Striking genus differences in urine microbiota composition between stone UR or tumor UR and controls. **a** The 44 different genus profiles of average relative abundance across three groups. C: control; S: stone UR; T: tumor UR. The dark green star indicates q < 0.01, light green star indicates q < 0.05, very light green star indicates p < 0.05, and gray star indicates q ≥ 0.05. **b** The relative abundance of 7 genera abundant in stone UR and tumor UR and 5 genera enriched in controls were exhibited with a box plot, respectively. **c** A network of Spearman’s correlation of the 44 significantly different genera among three groups. Blue edges, Spearman’s correlation coefficient < − 0.45; red edges, Spearman’s correlation coefficient > 0.45. **d** Heatmap of Spearman’s rank correlation coefficients of the relative abundance of different urine microbiota constituents at the genus level and 7 clinical indices. Genera that were enriched in controls are shown in blue. These asterisks indicate that the correlation is significant. GFR: glomerular filtration rate; ALP: alkaline phosphatase; DBIL: direct bilirubin; IBIL: indirect bilirubin; WBC: white blood cell count; UA: uric acid; RBC: red blood cell count

To explore the specific microbial signature of stone UR and tumor UR, we evaluated the relative abundance of taxa in three groups. Urine microflora composition in each sample from three groups at phylum and genus levels are shown in the Additional file 4: Figure S1A, D. Average composition of bacterial community at the phylum and genus levels are presented in Additional file 4: Figure S1B, E, among which the genus level list the top 35 bacterial and all the remaining low-abundance microflora are combined into “Others”. We found that *Proteobacteria* was the most abundant phylum in three groups, followed by *Bacteroidetes* (Additional file 4: Figure S1B). Compared with controls, stone UR and tumor UR individuals exhibited a significant increase in the phylum *Bacteroidetes* in urine (Additional file 4: Figure S1C). In accordance with the phylum level, the urine microbial compositions of the two types of obstructive UR patients were similar at the genus level, but both were different from that of the control group (Additional file 4: Figure S1E, F). It is noteworthy that there was a total of 44 bacteria with significant differences between any two of the three groups at the genus level (q < 0.01, Wilcoxon rank sum test, Fig. 2a). Twelve out of the 44 are displayed in Fig. 2b. *Elizabethkingia*, *Proteus*, *Sphingomonas,* *Pseudomonas, Acinetobacter*, *Sphingobacterium* and *Myroides* were overrepresented in the stone UR and tumor UR groups. In contrast, *Lactobacillus*, *Streptococcus*, *Gardnerella*, *Prevotella* and *Atopobium*, which were decreased in stone UR and tumor UR patients, were enriched in controls.

To further confirm the specific bacteria associated with obstructive UR, LEfSe was used, which identified 14 discriminative features, and their relative abundances significantly varied between stone UR individuals and controls, which was completely consistent with the result in Fig. 2a, as evaluated by the Wilcoxon rank sum test (Additional file 5: Figure S2A). *Curvibacter* was a newly found bacterium enriched in the tumor UR group, while *Escherichia* was found enriched in control group (Additional file 5: Figure S2B).

Evaluation of the connections among these different genera was performed by a Spearman correlation test. Significant positive correlations were found in genera enriched obstructive UR, such as for *Gluconacetobacter* and *Myroides* (R = 0.98, P = 1.24 × 10^−60^); *Gluconacetobacter* and *Sphingobacterium* (R = 0.94, P = 2.97 × 10^−40^); *Pseudomonas* and *Comamonas* (R = 0.85, P = 6.08 × 10^−24^); and so on. Likewise, positive correlations were also found in genera that were enriched in control subjects. More interestingly, the bacteria enriched in urine of the two obstructive UR patients were negatively correlated with those enriched in controls (Fig. 2c), such as *Myroides* and *Lactobacillus* (R = − 0.71, P = 7.93 × 10^−14^); *Sphingobacterium* and *Lactobacillus* (R = − 0.68, P = 3.45 × 10^−12^); *Elizabethkingia* and *Atopobium* (R = − 0.46, P = 1.44 × 10^−5^); and so on. We further explored the association of the urine microbiome with clinical manifestations (Fig. 2d) and found that there were some significantly negative correlations between the estimated glomerular filtration rate (eGFR) level and the microorganisms that were enriched in obstructive UR patients, such as *Pseudomonas, Methylobacterium, Elizabethkingia*, and so on. On the contrary, positive correlations were observed between the uric acid (UA) level and the genera that were enriched in obstructive UR patients, *su*ch as *Pseudomonas, Stenotrophomonas, Sphingomonas*, and so on.

### Classification of disease status using bacterial genus-level biomarkers

**Fig. 3:**
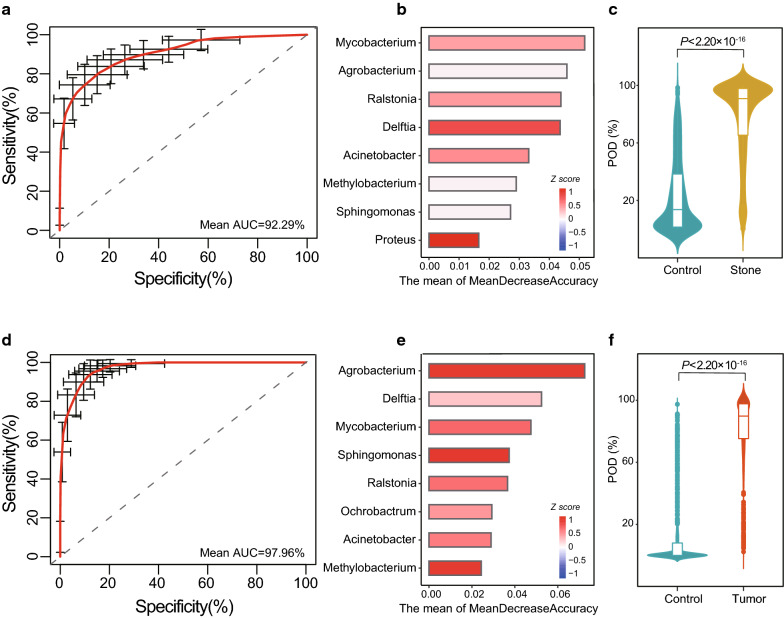
Microbial genus-based biomarkers of UR classification by a random forest model. **a** The average AUC value was calculated 100 times between stone UR and controls. **b**, **e** The optimal marker set was selected to distinguish stone UR or tumor UR from controls. The x-axis of the histogram represents the MDA. The color of each bar is the z value normalized by “heatmap” R package for the relative abundance of each bacterium. **c** The box figure shows the POD value between the stone UR and controls. **d** The average AUC reached 97.96% by the same method as above. **f** The POD value of the tumor UR group and controls

To explore the potential diagnostic value of the urine microbiome in stone UR and tumor UR, we constructed a random forest classifier to discriminate urinary retention samples from control samples. We only selected the microorganisms that were significantly enriched in stone UR and tumor UR to construct the classification models. Finally, 30 and 34 genera signatures were selected for further analysis for stone UR and tumor UR, respectively. The cross-validation error curve distribution was obtained from five trials of five-fold cross-validation. Both 8 biomarkers were selected as the optimal marker set to distinguish stone UR or tumor UR from the control group (Additional file 6: Figure S3A, B). The performance of these optimal marker models was assessed by 100 random ROC analyses, and the average AUC value achieved 92.29% between the stone UR and control group and 97.96% between the tumor UR and control group (Fig. 3a, d). The average MDA for the random 100 times of these optimal markers are shown in Fig. 3b and e. These results showed that 7 out of 8 bacteria were identical, which were used to distinguish stone UR or tumor UR from the control group, including *Mycobacterium*, *Agrobacterium*, *Ralstonia*, *Delftia*, *Acinetobacter*, *Methylobacterium* and *Sphingomonas*. Here, we further validated our previous finding that stone UR and tumor UR have approximate urine microbial background although they result from different obstruction causes. The POD value was significantly increased in stone UR group versus control group, and a similar trend was also found in tumor UR group (Fig. 3c, f; P< 2.2× 10^−16^ for both comparisons).

To select the optimal marker set of stone UR and tumor UR based on the urine microbiome, we used the genera with P < 0.05 which were measured by the Wilcoxon rank sum test. Only *Sediminibacterium* was selected from two different bacteria as the optimal marker after five repeated five-fold cross-validation trials Additional file 6: Figure S3C). The average AUC value was 58.44% after running 100 random ROC analyses (Additional file 6: Figure S3D). The MDA of *Sediminibacterium* is shown in Additional file 6: Figure S3E. The probability of tumor UR was significantly higher than stone UR in the tumor UR group (stone UR vs tumor UR, P= 1.00 × 10^−15^), which was consistent with these clinical data (Additional file 6: Figure S3F).

### Microbial functional altered in stone UR and tumor UR

**Fig. 4:**
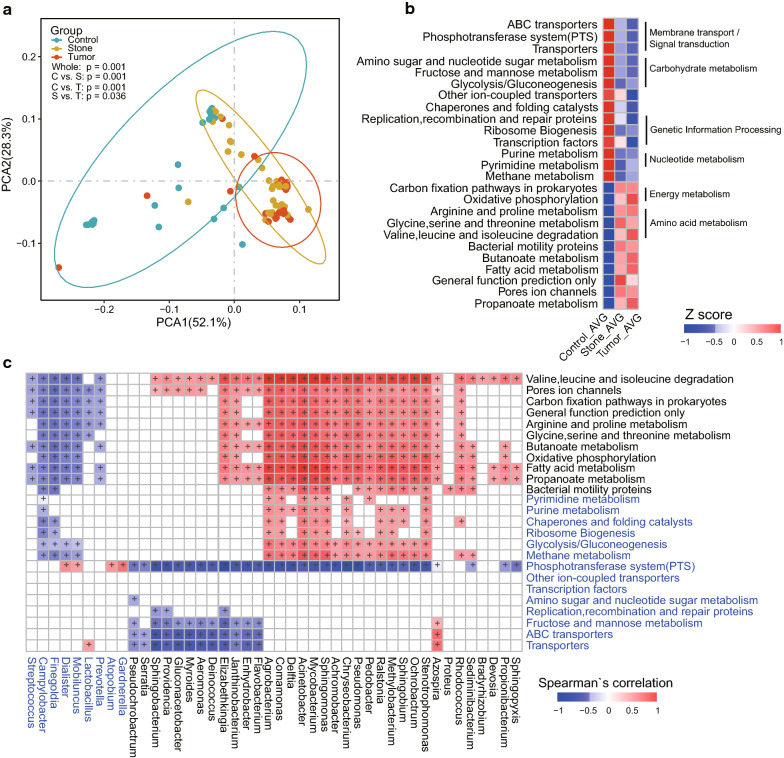
Microbial function profiles of the UR patients and control individuals. **a** The relative abundance of level 3 KEGG database pathways in all 82 samples is shown by PCOA. **b** A total of 25 KEGG pathways were significantly different among three groups, based on the average relative abundance of the KEGG (level 3) pathway. **c** Heatmap of Spearman`s correlation of the 44 significantly different genera and 25 significantly different pathways among three groups (|r| > 0.3). The genera or pathways that were enriched in the controls are shown in blue. These “+” indicate that the correlation is significant

To explore whether the function of the urine microbiome of stone UR and tumor UR has changed, we used PICRUSt to predict the functional components of the 16S rRNA gene sequencing data of all samples. The OTU profile of these three groups was aligned to level 3 of the KEGG database, and COG abundance was calculated (Additional file 7: Table S4, Additional file 8: Table S5). PCOA based on the KEGG pathways showed that the control group was strikingly separated from stone UR and tumor UR groups (Fig. 4a; PERMANOVA, pseudo-F statistic: 13.05, P= 1.00 × 10^−3^), which was consistent with the result based on COG categories (Additional file 9: Figure S4A; PERMANOVA, pseudo-F statistic: 11.97, P= 1.00 × 10^−3^). We found that both obstructive UR groups were significantly different from control group, while the functional structure between stone UR and tumor UR was similar (Fig. 4b). Twenty-five KEGG pathways were differentially enriched between each two of the three groups (adjusted p-value < 0.01, Wilcoxon rank sum test). There were 14 pathways involved in membrane transport, signal transduction, genetic information processing, carbohydrate metabolism and nucleotide metabolism, such as that for purine and methane, which were significantly reduced in the stone UR group and tumor UR group. We observed 11 pathways that were increased in the stone UR and tumor UR groups, including amino acid metabolism and energy metabolism. Intriguingly, the abundance of pathways associated with membrane transport functions, such as ABC transporters and the phosphotransferase system (PTS), was negatively correlated with these genera enriched in stone UR and tumor UR groups. In accordance with the KEGG function result, most of the energy metabolism, carbohydrate metabolism and amino acid metabolism pathways had changed in the COG annotation (Additional file 9: Figure S4B). There was a strong positive correlation between the significantly enriched functions in both obstructive UR groups and their enriched genera (Fig. 4c). For instance, *Pseudomonas*, *Acinetobacter* and *Sphingomonas* were significantly related to valine, leucine and isoleucine degradation and glycine, serine and threonine metabolism. These amino acids may be the product of bacteria as *Corynebacterium glutamicum* can produce amino acids on a large scale reported previously [34]. ABC transporters, the PTS, transporters and other ion-coupled transporters that were significantly enriched in controls were mainly negatively correlated with patients with UR microbial features. The same trend was found in the COG function analysis with these significantly different bacteria (Additional file 9: Figure S4C).

## Discussion

This current study aims to analyze the microbial spectrum of two common types of obstructive urinary retention using 16S rRNA gene sequencing technology, a widely recognized technique that can depict the landscape of microorganisms. Our results showed that the microbial diversity of stone UR and tumor UR patients increased significantly compared with that of controls, while there was no obvious difference between these two types of obstructive UR. Changes in microbiome diversity of urinary system disorders have not been consistently. Previous studies have found increased microbial diversity in UUI [35] and chronic prostatitis patients [17] and decreased bacterial diversity in subjects with IC [16]. In addition, some urinary system diseases, such as overactive bladder [36], had no significant changes in microbial diversity. The increased community richness and diversity of the urine microbiome in stone UR and tumor UR groups is consistent with previous findings [37, 38]. Although the urine microbiota profile differs among individuals, which mainly due to genetic background, age, dietary habits and lifestyle, samples with same clinical phenotype was clustered in β-diversity analysis for their common urine microbial composition.

We observed a dramatic shift in the components of the urine microbiome in patients with two types of obstructive UR, however the urine microbiota feature of the stone UR and tumor UR patients were similar. A gram-negative genus, *Pseudomonas*, is the second most common infection in hospitalized patients. In addition, *Pseudomonas* is known to be associated with a wide range of urinary tract diseases [39–41]. In 2015, Barr-Beare et al. not only detected *Pseudomonas* by sequencing from a urinary stone but also isolated this bacterium from stone culture [39]. Used 454 sequencing technology, Xu et al. detected that *Pseudomonas* was the dominant genus in some patients with urothelial carcinoma [42]. This finding agreed with previous studies that demonstrated that *Pseudomonas* spp. were enriched in stone UR and tumor UR patients. *Acinetobacter* is a complex genus, the virulence factors of *Acinetobacter baumannii* have been proofed to be involved in epithelial cell adhesion and invasion, biofilm formation [43]. A study profiling the urinary microbiome in man with calcium-based kidney stone by Xie et al., which shown the most differentially represented taxa at genus level was *Acinetobacter* and enrichment in kidney stone patients [44]. Furthermore, *Acinetobacter* was an abundant genus in urothelial carcinoma patients and in male patients with bladder cancer in China [42, 45]. *Sphingobacterium* and *Sphingomonas*, which were also elevated in the tumor UR group, are morbidity-inducing urine bacteria that cause bladder cancer, prostate cancer and BPH [38, 46]. In addition, we found two unusual bacteria. The first is *Myroides*, which was the cause of an outbreak of UTI in a Tunisian hospital [47]. Almost all patients infected with *Myroides* spp. had a urinary stone or urinary neoplasms. *Elizabethkingia* was the second genus, and in 2017, a 25-year-old woman was the first individual reported to have a UTI caused by *Elizabethkingia* and to have difficulty urinating [48]. On ground of our findings and previous reports, we assume that there is a strong association between obstructive UR and UTI, but it is not clear whether there is a causal relationship between them and what role microorganisms play. Furthermore, our results also indicated that a complex bacterial community dominated by *Lactobacillus*, *Prevotella*, *Streptococcus* and *Gardnerella* exists in the urinary tract of healthy people. These microorganisms have been speculated in previous studies to be mandatary to maintain a healthy status in the urinary system [11, 13, 20, 49, 50]. *Lactobacillus* plays an important role in the human body because it can protect the host from potential pathogens and maintaining urinary health [51]. *Streptococcus* has been found repeatedly in the urine of healthy men [52, 53]. A 2018 study showed that increased of *Streptococcus* in healthy bladder midstream urine versus the bladder cancer [37]. In addition, the microbial population profile of another urologic malignancy, prostate cancer, also found significant enrichment of *Streptococcus* in the non-tumor tissue [54]. Xu et al. detected the abundance of *Streptococcus* was near zero in most control patients but significantly elevated in urothelial carcinoma patients, however, which is the opposite of what was observed in our study [42]. Therefore, needed more research to elucidate the association between *Streptococcus* and the cancer or health status. It is worth mentioning that these beneficial microbial enriched in control group were negatively correlated with the pathogenic bacteria in the stone UR and tumor UR groups but positively correlated internally. Of interest, products of some bacteria might inhibit the activity of others. For example, lactic acid produced by *Lactobacillus* showed good inhibition activity against *E. coli* [55].

Stone UR and tumor UR microbial anomalies were characterized by altered relative abundance in 44 genera. The combination of the optimal marker taxa that distinguished stone UR or tumor UR patients from healthy controls had high accuracies of 92.29% and 97.96%, respectively. It suggests that these differentially present microbial communities may be a potentially effective tool for predicting stone UR or tumor UR, and further combination with clinical information may enhance the identification capability. Further detailed studies are needed to obtain better classifiers to distinguish stone UR and tumor UR in the future.

Along with the altered composition of the urine microbiome, their functional change was also predicted by PICRUSt. Biosynthesis and metabolism of some carbohydrates and nucleotides were depleted in obstructive UR, such as amino and nucleotide sugar metabolism, fructose and mannose metabolism and glycolysis/gluconeogenesis, which are essential for the host. Pathway analysis also showed a decline in the capacity for membrane transport and signal transduction, indicating impaired membrane permeability or ureteral obstruction. Ureteral obstruction leads to a gradual decrease in renal excretion with decreased eGFR levels [56]. In our cohort, the majority of pathogenic genera in stone UR and tumor UR were negatively correlated with eGFR levels, suggesting a potential link between these microbes and disease severity. Similarly, many pathogens of UR were significantly positively correlated with UA content. Clinical knowledge suggests that the elevated UA content is a result of host purine metabolism disorder. Purine metabolism was reduced in patients with obstructive UR, as expected, hinting at a potential role of urine microbes in causing nucleotide metabolism dysfunction. Although the purine metabolism ability was weakened in two UR groups, UA content could not be excreted due to obvious impairment of membrane transport, which showed that UA levels of these two obstructive UR patients were higher than those of control individuals (Table 1).

There are still several limitations in our study to be addressed. First, it is difficult to determine the causal relationship between the microflora and obstructive UR. Therefore, prospective follow-up studies with larger sample sizes and experimental studies are needed to detect the role of the microbiome in obstructive UR progression and development. Second, our pathway and function characterizations were inferred by PICRUSt based on the 16S rRNA sequence. In the future, the combination of metagenomics and metaproteomics may reveal more accurate microbial community composition and function.

## Conclusions

In summary, we characterized the microbiome profiles of two obstructive UR, stone UR and tumor UR, in comparison with control subjects. Our study suggested that the urine microbiome may be associated with obstructive UR, while the cause-effect relationship remains to be elucidated. Some bacteria can be used to discriminate obstructive UR patients from healthy individuals with high accuracy. A better understanding of the role of the urinary microflora in obstructive UR would help urologists make more sensible choices in clinical identification and formulate personalized microbial intervention measures.

## Supplementary information

**Additional file 1: Table S1.** Data production of 84 samples in three groups.

**Additional file 2: Table S2.** The relative abundance of the OTU profile and taxonomy.

**Additional file 3: Table S3.** PERMANOVA tests of the urine microbiota at different dissimilarity distances.

**Additional file 4: Figure S1.** Phylogenetic profiles of urine microbes among stone UR and tumor UR and controls. (A, D) The microflora composition abundance of each sample at the phylum level (A) and genus level (D). (B, E) Composition of the urine microbiota at the phylum level (B) and genus level (E) among three groups. (C) The 5 different phylum profiles of average relative abundance across three groups tested by the Wilcoxon rank sum test. The dark green star indicates q < 0.01, light green star indicates q<0.05, very light green star indicates p < 0.05, and gray star indicates q ≥ 0.05. (F) PCoA analysis based on genus level of for two types UR and healthy controls.

**Additional file 5: Figure S2.** LEfSe analyses of the urine microbiomes of stone UR and tumor UR patients compared to those of controls. (A) The LDA bar graphs show differential abundance between stone UR and controls. (B) The LDA bar graphs show differential abundance between tumor UR and controls.

**Additional file 6: Figure S3.** A random forest model was established to distinguish stone UR from tumor UR based on microbial genus. (A, B, C) 8, 8 and 1 genus were selected as the optimal marker set for the three ‘randomForest’ model respectively (A: controls vs stone UR; B: controls vs tumor UR; C: stone UR vs tumor UR). (D) The average AUC value after 100 ROC measurements to distinguish two types of UR. (E) The MDA of this marker between stone UR and tumor UR. (F) The probability of tumor UR value between two types of obstructive UR.

**Additional file 7: Table S4.** The relative abundance of level 3 KEGG pathway profiles predicted by PICRUSt.

**Additional file 8: Table S5.** The relative abundance of level 2 COG functions predicted by PICRUSt.

**Additional file 9: Figure S4.** Microbial function profiles of these UR patients and the control subjects. (A) PCOA analysis for the relative abundance of level 2 COG categories of all participants. (B) A total of 18 COG functions were significantly different among three groups. (C) Heatmap of Spearman’s correlation of the 44 significantly different genera and 18 significantly different pathways among three groups. The genera or pathways that were enriched in controls are shown in blue.

## Data Availability

The datasets used and/or analyzed in this study are available from the corresponding author on reasonable request.

## Abbreviations

UR: Urinary retention
BPH: Benign prostatic hyperplasia
UI: Urinary incontinence
UTI: Urinary tract infection
UUI: Urgency urinary incontinence
IC: Interstitial cystitis
CP/CPPS: Chronic prostatitis/chronic pelvic pain syndrome
USD: Urinary stone disease
EQUC: Expanded quantitative urine culture
OTU: Operational taxonomic unit
QIIME: Quantitative Insights Into Microbial Ecology
LefSe: Linear discriminant analysis for effect size
PICRUSt: Phylogenetic Investigation of Communities by Reconstruction of Unobserved States
PCoA: Principal coordinate analysis
PERMANOVA: Permutational multivariate analysis of variance
ROC: Receiver operating characteristic curve
AUC: Area under the curve
MDA: Mean decrease accuracy
POD: Probability of disease
KEGG: Kyoto Encyclopedia of Genes and Genomes
COG: Orthologous Groups of proteins
PTS: Phosphotransferase system
