# Supplementary material for: Dysbiosis of urine microbiota in obstructive urinary retention patients revealed by next-generation sequencing
Source: Ann Clin Microbiol Antimicrob. 2021 Jan 6;20:2. doi: 10.1186/s12941-020-00408-5 (PMC7789751; doi:10.1186/s12941-020-00408-5)
Supplement: Supplementary file 3 — Additional file 3: Table S3. PERMANOVA tests of the urine microbiota at different dissimilarity distances. [file 12941_2020_408_MOESM3_ESM.docx]

**Table S3. PERMANOVA tests of the urine microbiota at different dissimilarity distances.**

|  | **Unweighted Unifrac** | | | **weighted Unifrac** | | | **Bray-Curtis** | | |
| --- | --- | --- | --- | --- | --- | --- | --- | --- | --- |
| **Category** | **F** | **R^2^** | **P-value** | **F** | **R^2^** | **P-value** | **F** | **R^2^** | **P-value** |
| **All** | 11.88 | 0.23 | 0.001 | 9.39 | 0.19 | 0.001 | 5.97 | 0.13 | 0.001 |
| **Control (n=25)**  ***vs***  **Stone UR (n=32)** | 15.75 | 0.22 | 0.001 | 12.24 | 0.18 | 0.001 | 6.37 | 0.10 | 0.001 |
| **Control (n=25)**  ***vs***  **Tumor UR (n=25)** | 14.99 | 0.24 | 0.001 | 12.39 | 0.21 | 0.001 | 10.02 | 0.17 | 0.001 |
| **Stone UR (n=32)**  ***vs***  **Tumor UR (n=25)** | 1.38 | 0.02 | 0.18 | 2.02 | 0.04 | 0.08 | 1.68 | 0.03 | 0.10 |
