# Supplementary figures and images for: Dysbiosis of urine microbiota in obstructive urinary retention patients revealed by next-generation sequencing
Source: Ann Clin Microbiol Antimicrob. 2021 Jan 6;20:2. doi: 10.1186/s12941-020-00408-5 (PMC7789751; doi:10.1186/s12941-020-00408-5)

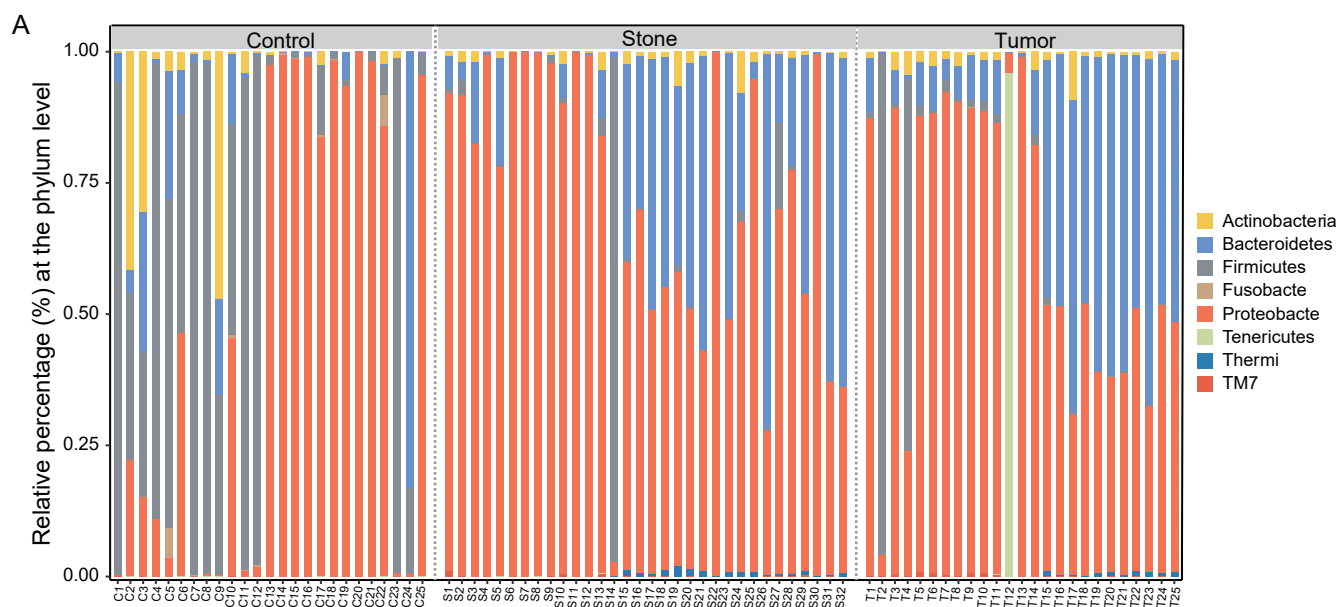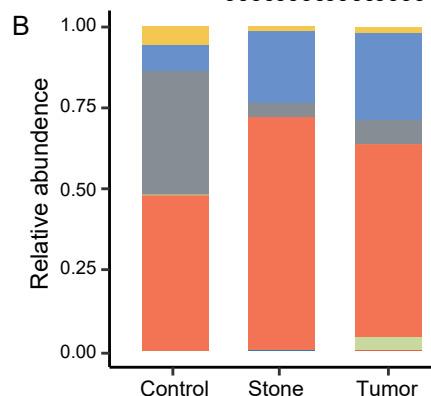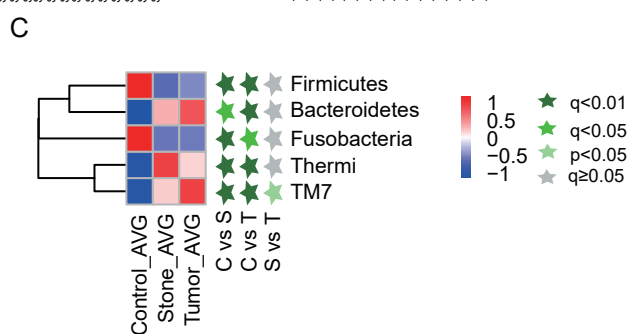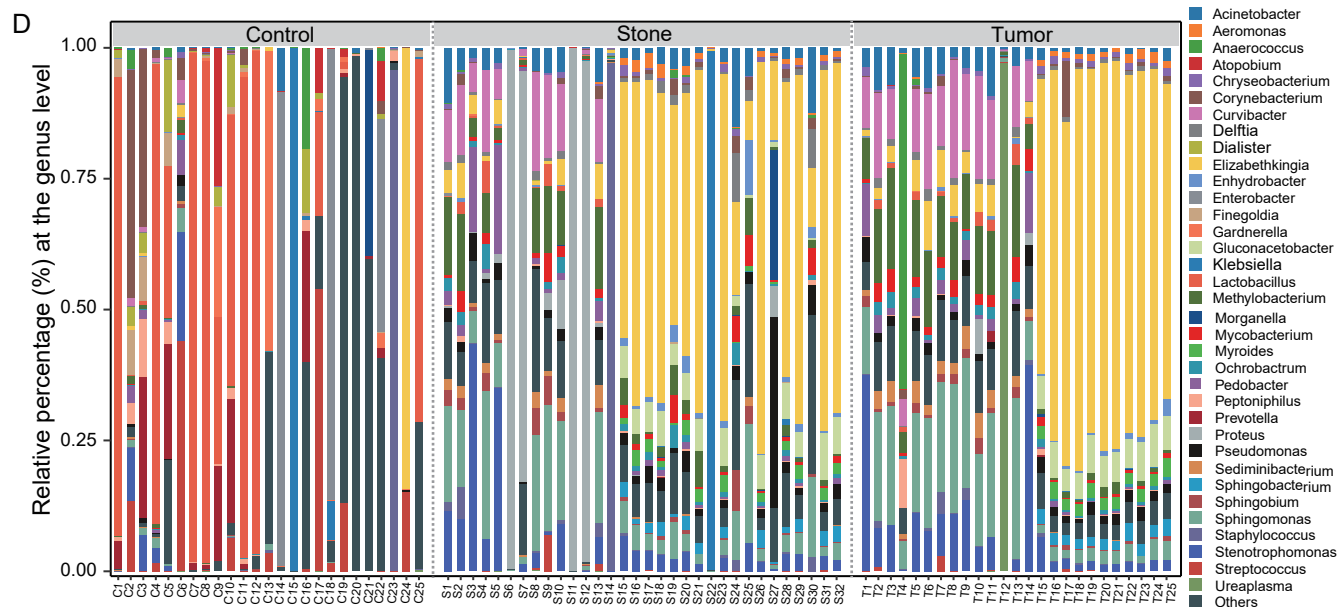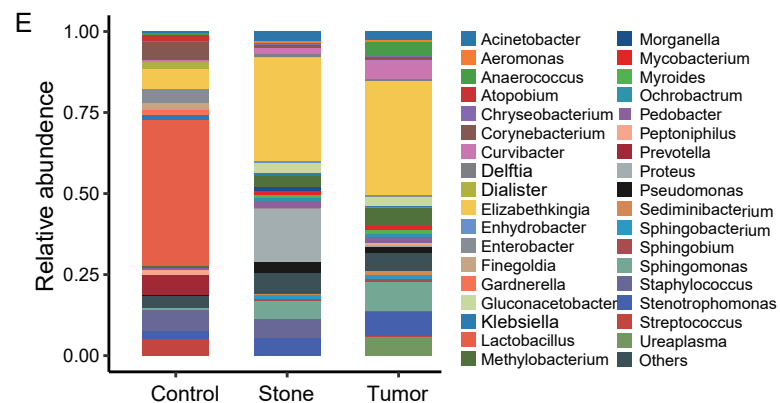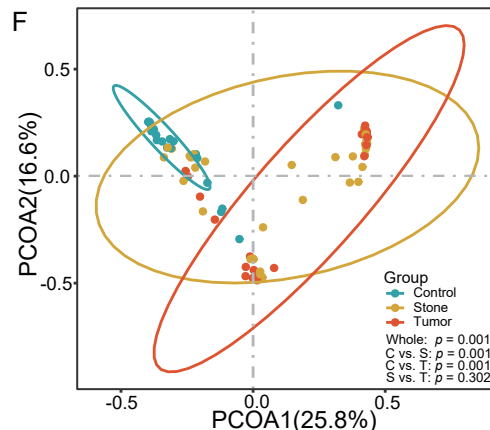

Supplement: Supplementary file 4 — Additional file 4: Figure S1. Phylogenetic profiles of urine microbes among stone UR and tumor UR and controls. (A, D) The microflora composition abundance of each sample at the phylum level (A) and genus level (D). (B, E) Composition of the urine microbiota at the phylum level (B) and genus level (E) among three groups. (C) The 5 different phylum profiles of average relative abundance across three groups tested by the Wilcoxon rank sum test. The dark green star indicates q < 0.01, light green star indicates q<0.05, very light green star indicates p < 0.05, and gray star indicates q ≥ 0.05. (F) PCoA analysis based on genus level of for two types UR and healthy controls. [file 12941_2020_408_MOESM4_ESM.pdf]

A

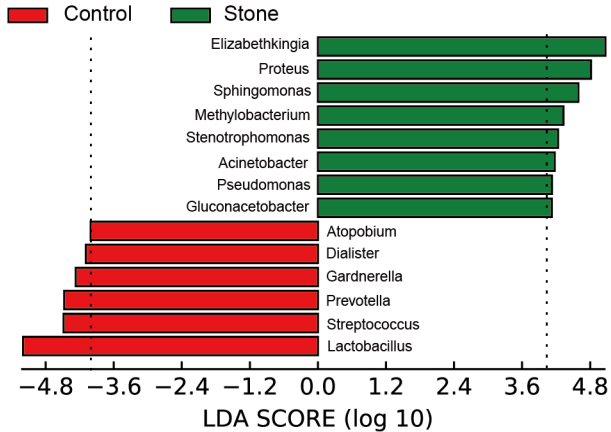

B

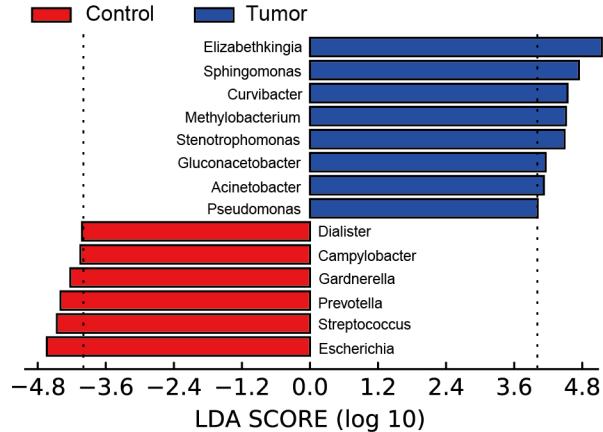

Supplement: Supplementary file 5 — Additional file 5: Figure S2. LEfSe analyses of the urine microbiomes of stone UR and tumor UR patients compared to those of controls. (A) The LDA bar graphs show differential abundance between stone UR and controls. (B) The LDA bar graphs show differential abundance between tumor UR and controls. [file 12941_2020_408_MOESM5_ESM.pdf]

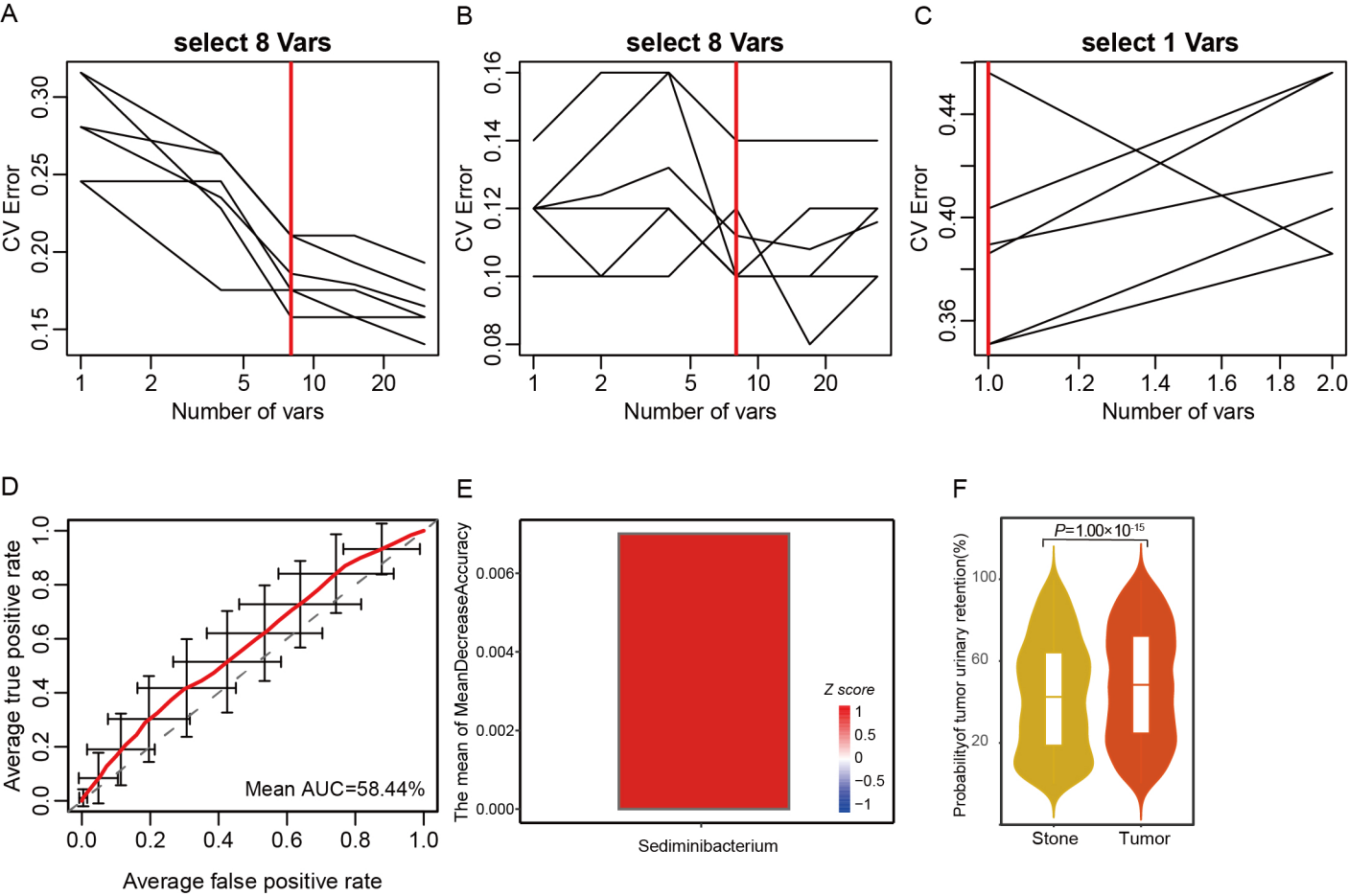

Supplement: Supplementary file 6 — Additional file 6: Figure S3. A random forest model was established to distinguish stone UR from tumor UR based on microbial genus. (A, B, C) 8, 8 and 1 genus were selected as the optimal marker set for the three ‘randomForest’ model respectively (A: controls vs stone UR; B: controls vs tumor UR; C: stone UR vs tumor UR). (D) The average AUC value after 100 ROC measurements to distinguish two types of UR. (E) The MDA of this marker between stone UR and tumor UR. (F) The probability of tumor UR value between two types of obstructive UR. [file 12941_2020_408_MOESM6_ESM.pdf]

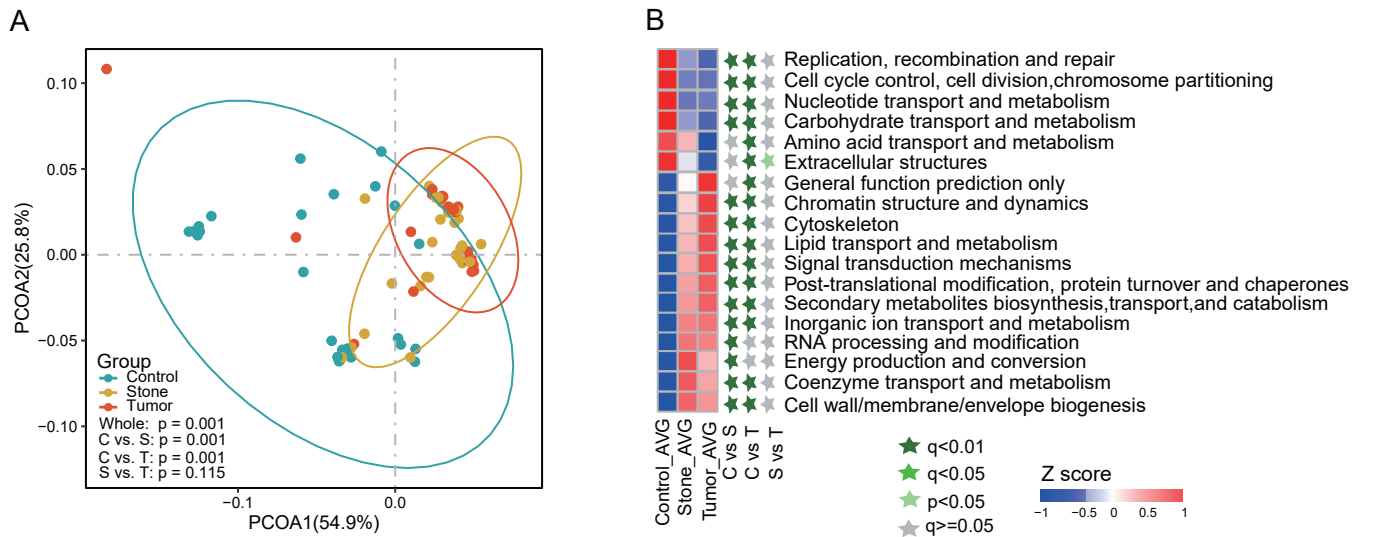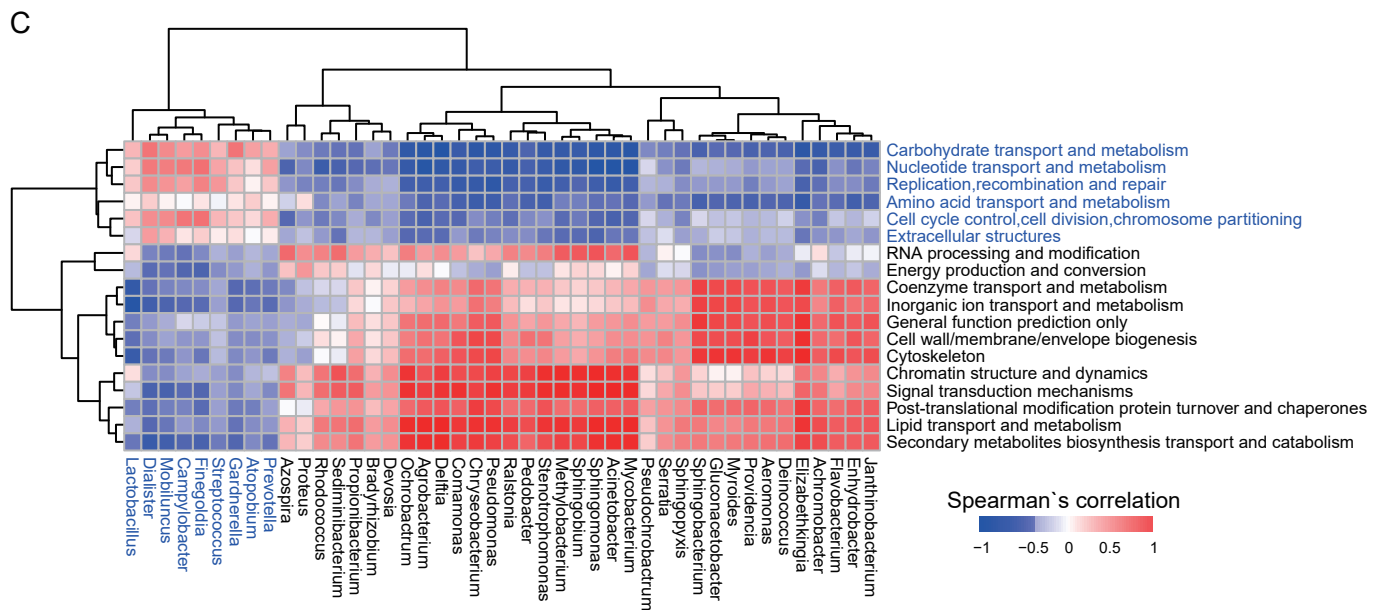

Supplement: Supplementary file 9 — Additional file 9: Figure S4. Microbial function profiles of these UR patients and the control subjects. (A) PCOA analysis for the relative abundance of level 2 COG categories of all participants. (B) A total of 18 COG functions were significantly different among three groups. (C) Heatmap of Spearman’s correlation of the 44 significantly different genera and 18 significantly different pathways among three groups. The genera or pathways that were enriched in controls are shown in blue. [file 12941_2020_408_MOESM9_ESM.pdf]
